# Non-adherence to growth monitoring and promotion sessions amongst caregivers of children under 5 years in Polokwane Municipality, Limpopo province

**DOI:** 10.4102/safp.v65i1.5523

**Published:** 2023-02-20

**Authors:** Mabitsela H. Mphasha, Matjie Rapetsoa, Nkhaviso Mathebula, Kamogelo Makua, Sanele Mazibuko

**Affiliations:** 1 Department of Human Nutrition and Dietetics, Faculty of Healthcare Sciences, University of Limpopo, Polokwane, South Africa

**Keywords:** growth monitoring and promotion, non-adherence, caregivers, stunting, sessions

## Abstract

**Background:**

Child growth is crucial for nutritional and health status; poor growth may result in stunting. South Africa experiences a high prevalence of stunting, micronutrient deficiencies and late identification of growth faltering. Non-adherence to growth monitoring and promotion (GMP) sessions remains a challenge and caregivers contribute to non-adherence. Therefore, this study explores factors affecting the non-adherence of GMP services.

**Methods:**

Qualitative approach and phenomenological exploratory study design were used. One-on-one interviews were conducted with 23 participants conveniently sampled. Sampling size was dependent on data saturation. Voice recorders were used to capture data. Tesch’s eight steps, inductive, descriptive and open coding techniques were used to analyse data. Measures of trustworthiness were ensured through credibility, transferability, dependability and confirmability.

**Results:**

Participants indicated non-adherence to GMP sessions because of a lack of knowledge of the importance of adherence and poor service by healthcare workers, which includes long waiting hours. Inconsistent availability of GMP services at healthcare facilities and growth of the firstborn children with non-adherence to GMP sessions are factors influencing participants’ adherence. A lack of transportation and lunch money also contributed to non-adherence to sessions.

**Conclusion:**

A lack of knowledge of the importance of adherence to GMP sessions, long waiting hours and inconsistent availability of GMP services at facilities contributed enormously to non-adherence. Therefore, the Department of Health must ensure consistent availability of GMP services to demonstrate the importance and enable adherence. Healthcare facilities should reduce waiting hours to minimise the need for lunch money, and service delivery audits should be conducted to identify other factors contributing to non-adherence to address these.

**Contribution:**

Primary health care providers should conduct service delivery audits and internal surveys to identify factors that contribute to non-adherence in order to introduce measures to address them.

## Background

Child growth is important in assessing nutritional and health status, wherein weight is used to assess the growth.^1^ However, child growth and development are a public health problem, with the recent worldwide prevalence of stunting estimated at 144 million children under the age of five (approximately 21.3%).^2^ Stunting is most prevalent in the poorest and food-insecure nations, including middle-income countries.^2^ A Ghanaian study amongst children under five years found that 19.0% were stunted, 5.0% wasted and 11.0% underweight.^3^ Ethiopia has the highest numbers of stunted, underweight and wasted children in the sub-Saharan African region.^4^ The 2020 South African Child Gauge, focusing on nutrition and food security, reported that South Africa (SA) is experiencing a high prevalence of stunting rates, micronutrient deficiencies and overweight and obesity, despite SA being classified as an upper-middle-income country.^5^ It has been reported that 27.0% of children were stunted in 2016 whilst 2.0% were wasted.^6^ The latest available data from 2016 shows that there are 1 564 000 children in SA who are already stunted and will not reach their full growth potential.^6^ It is projected that SA will have an estimated 1.7 million stunted children in 2025.^5^

South Africa adopted the use of growth monitoring and promotion (GMP) in minimising the prevalence of malnutrition, infectious diseases and death amongst children under the age of five years.^7^ The GMP involves regular measuring and plotting of weight of children under five years using anthropometric measurements in a growth chart within specific intervals, including comparing growth curves in comparison to standards of the World Health Organization (WHO).^1,8^ Based on the outcomes, healthcare providers provide counselling to mothers and caregivers to take corrective steps to improve the growth of the child^9^ before the child’s nutritional status degenerates to full-blown malnutrition.^8^ In addition, it provides a warning signal for timely and appropriate action in incorporating, identifying and correcting growth faltering. Usually, the GMP services are delivered together with other child health services such as vaccinations and supplementation.^10^ Hence, it is essential in caring for children and promoting child development^3^ and is linked with long-term health, economic and social benefits.^11^

Caregivers of children under the age of five years were found to be contributing to non-adherence of GMP sessions because children are dependent on them to access health facilities.^12^ Factors such as financial constraints and problems relating to the health system itself, such as lack of parking and limited weekend or evening clinics, contribute to non-adherence.^4,13^ Knowledge of the importance of GMP amongst caregivers of children under five years has been found to be critical for adherence and health outcomes of children.^14^ A Kenyan study revealed that the lack of caregivers’ indigenous understanding of child health and growth led to non-adherence.^14^ The lack of support by husbands or partners of mothers regarding overall child feeding is a challenge, which impacts adherence to GMP sessions.^4^ It has been reported that limited or absence of community awareness campaigns, including unsatisfactory counselling from healthcare providers regarding child nutrition as GMP programme, contribute significantly to non-adherence. Caregivers of children also indicated that the way healthcare professionals look at them, including the method of weighing their children, contributes to non-adherence.^14^ Insufficient or lack of community involvement, including lack of appropriate support from healthcare providers, poor referral systems and monitoring, contributes to non-adherence.^4^ So far, efforts were taken to promote adherence to GMP services, yet non-adherence is still a public health problem which compromises the health of children. Therefore, this particular study is aimed at exploring factors affecting the non-adherence of GMP services in Polokwane.

## Research methodology

### Research method and design

A qualitative method was adopted in conducting this study, including the use of a phenomenological exploratory study design. One-on-one interviews were conducted with participants who described factors affecting non-adherence to GMP sessions among caregivers of under-five children.

### Study site

This study was conducted in the rural part of Polokwane Municipality, specifically Sebayeng Clinic, in the Dikgale cluster of Polokwane Municipality. The study site was selected to understand challenges related to non-adherence to GMP sessions. The GMP services are offered twice a week at this clinic, mainly by primary health care nurses and dietititians. Students from other departments, such as nursing at the University of Limpopo, also do practicals at this clinic by offering GMP services amongst others.

### Sampling and participants

The target population for this study were caregivers of children under the age of five years attending GMP services in Sebayeng Clinic. Caregivers in this context refers to mothers and legal guardians who assume the primary responsibility for looking after children under the age of five years. Population size of this study was estimated at 300 caregivers. Therefore, this particular clinic was selected because it has many caregivers of children under the age of five years, which made it easier to obtain participants who met inclusion criteria for this study. Inclusion criteria in this study were only caregivers of children under the age of five years with records of missed clinic appointments or GMP sessions, because they could relay the factors influencing them towards adherence or non-adherence to GMP sessions.

Twenty-three participants were conveniently sampled from the target population of this study. Data saturation was used to determine the sample size. The data saturation was reached at 20 participants, however an additional three participants were sampled to check for additional new information. However, no new information was added, confirming saturation and therefore discontinuation of data collection.

### Data collection

One-on-one interviews were used to collect data from participants. Voice recorders were used to capture the interviews and participants were alerted when switched on and off to avoid capturing information participants wish to say off-record. The interviews were conducted by student dietitians doing community nutrition practicals at the selected clinic. Only recorded data were used in this study. The interviews were conducted in Sepedi because the people in the area were Sepedi-speaking persons. The main question that was asked to participants at the opening of interviews was, ‘[*k*]indly describe factors that influenced adherence or non-adherence to scheduled clinic visits’. The interview guide was used during interviews; however, follow-up questions or probing was performed based on the responses of participants for each question.

#### Trustworthiness

For more details, please see Table 1.^15^

**TABLE 1:** Measures of trustworthiness.

Strategy	Criteria	Applicability
Credibility	Prolonged engagement	Researchers responsible for data collection were student dietitians conducting community nutrition practicals at Sebayeng clinic. The practical included offering GMP services to caregivers. Therefore, researchers were familiar and known in the area. In addition, researchers collected data from each participant for about 15–45 min to extract more information from participants.
	Member checks	Immediately after the conclusion of each interview, a summary was provided to the participant for confirmation of findings.
Transferability	Data saturation	Participant number 20 added no new data; extra three participants were further sampled for confirmation of data saturation. No new information was added; data collection was therefore discontinued.
	Thick description	A clear description of study setting, how participants were sampled, and data collected was provided in detail. Therefore, this shall permit other researchers to transfer findings.
Confirmability	Peer review	Three researchers collected and analysed the data independently, developed themes and sub-themes independently and further met for consensus. Thereafter, they met with supervisors and agreed on themes and sub-themes.
	Reflexitivity	The researchers remained neutral during the process of data collections and used probing and reflexitivity to get more data from participants by using statements such as, ‘so what you are actually saying is …’
Dependability	Dense description of research methods	The researchers depended on clear and detailed description of research methods, including the use of participants’ quotation in presenting results.
	Audit trail	Researchers who collected data explained the process of data collection and analysis to supervisors. Consensus on themes and sub-themes were reached. Data to be stored for the next 5 years in lockable files.
	Reliance on data collection tools and supervisors	Researchers relied on voice recorder to capture interviews and experiences of supervisors.

*Source:* Ramathuba DU, Ndou H. Ethical conflicts experienced by intensive care unit health professionals in a regional hospital, Limpopo province, South Africa’, Health SA. 2020;25(0):1-9. https://doi.org/10.4102/hsag.v25i0.1183

GMP, growth monitoring and promotion.

### Data analysis

All interviews were audio-taped and transcribed. Interviews were conducted in Sepedi; therefore, they were translated into English before analysis. Services of a language translator were procured to minimise data loss. Thereafter, all researchers involved in data collection, including supervisors, analysed verbatim transcripts independently. A meeting with supervisors was arranged, and an agreement on themes and sub-themes was reached. Participants’ direct quotations are captured in italic format to support findings. Data were analysed using the eight steps of Tesch’s open coding qualitative data analysis method by Creswell,^16^ as shown in Table 2.

**TABLE 2:** Tesch’s eight steps of open coding.

Steps	Applicability
Step 1 – Reading through the data	The researcher got a sense of the whole by reading all the verbatim transcripts carefully and repeatedly. This gave ideas about the data segments and their meaning. The researchers understood transcripts and jotted down ideas.
Step 2 – Reduction of the collected	The researcher scaled down the data collected to codes, based on the existence or frequency of concepts used in the verbatim transcriptions. The researcher then listed all topics that emerged during the scaling down and grouped similar topics together; those that did not have association were clustered separately.
Step 3 – Asking questions about the meaning of the collected data	Questions emerged about transcriptions of the interview, based on the codes which existed from the frequency of the concepts. Questions included, ‘what is this about?’ and ‘what is the underlying meaning?’
Step 4 – Abbreviation of topics to codes	The researchers abbreviated topics that emerged as codes. These codes were written next to the appropriate segments of the transcription. All these codes were written on the margins of the paper.
Step 5 – Development of themes and sub-themes	The researcher developed themes and sub-themes from coded data and the associated texts and reduced the total list by grouping topics that relate to one another to create the meaning of the themes and sub-themes.
Step 6 – Compare the codes, topics and themes for duplication	The researchers reworked from the beginning to check the work for duplication. There was no necessity to refine the codes, topics and themes.
Step 7 – Initial grouping of all themes and sub-themes	The data belonging to each theme were assembled in one column and preliminary analysis was performed, which was followed by the meeting between the researcher, supervisors and co-coder to reach consensus on themes and sub-themes that each one had come up with independently.
Step 8 – Recoding if necessary	There was no need for recoding.

*Source:* Creswell JW. Qualitative inquiry and research design: Choosing among five approaches. 3rd ed. Thousand Oaks, CA: Sage; 2013.

### Ethical considerations

Turfloop Research Ethical Committee (TREC) permitted the study to be conducted with clearance certificate number TREC/238/2021:UG. Limpopo Department of Health (DOH) and clinic manager granted permission to conduct the study. Signed informed consent was obtained from participants, confirming their voluntary participation. Participants were briefed about their rights to withdraw from the study at any stage without penalty. Privacy and confidentiality of the participants’ data were also maintained.

## Results

### Demographic information

The demographic information describes participants that were interviewed in this study. The demographic information of the participants is presented in Table 3.

**TABLE 3:** Socio-demographic profile of participants.

Participants number	Gender	Age of participants (years)	Marital status	Employment status	Educational level	Age of children
1	Female	40	Single	Unemployed	Tertiary	24 months
2	Female	35	Married	Unemployed	Tertiary	6 months
3	Female	31	Single	Employed	Secondary	18 months
4	Female	55	Married	Unemployed	Primary	6 months
5	Female	23	Single	Employed	Secondary	24 months
6	Female	37	Single	Unemployed	Secondary	4 months
7	Female	44	Single	Unemployed	Secondary	14 weeks
8	Female	23	Single	Unemployed	Tertiary	18 months
9	Female	46	Married	Unemployed	Primary	3 months
10	Female	18	Single	Unemployed	Secondary	6 months
11	Female	25	Married	Employed	Tertiary	36 months
12	Female	50	Single	Unemployed	Primary	36 months
13	Female	24	Single	Unemployed	Secondary	36 months
14	Female	27	Single	Employed	Tertiary	12 months
15	Female	30	Married	Employed	Secondary	15 months
16	Female	33	Married	Employed	Secondary	50 months
17	Female	38	Single	Unemployed	Secondary	30 months
18	Female	24	Single	Unemployed	Secondary	24 months
19	Female	31	Single	Employed	Tertiary	28 months
20	Female	29	Single	Employed	Primary	14 months
21	Female	44	Married	Unemployed	Tertiary	46 months
22	Female	35	Married	Employed	Secondary	52 months
23	Female	35	Single	Employed	Primary	24 months

Note: This table shows that out of 23 participants, 17 were between the ages of 20 and 40 years, 17 were married, 13 were unemployed and 11 had secondary education, whilst 15 had children between the ages of 6 and 24 months.

Three themes emerged from data analysis (as shown in Table 4): (1) knowledge related to GMP, (2) healthcare services and (3) socio-economic factors. Moreover, sub-themes were developed from each theme.

**TABLE 4:** Themes and sub-themes.

Themes	Sub-themes
1. Knowledge related to GMP	1.1. Knowledge of the importance of GMP 1.2. Lack of knowledge of the importance of adherence to GMP sessions 1.3. Lack of knowledge on the interpretation of growth charts
2. Healthcare services	2.1. Poor services by healthcare providers 2.2. Attitudes of healthcare providers
3. Socio-economic factors	3.1. Work-related factors 3.2. Lack of transportation and lunch money

GMP, growth monitoring and promotion.

#### Theme 1: Knowledge related to growth monitoring and promotion

Findings from this study revealed knowledge of the importance of GMP, including the benefits associated with adherence to GMP sessions. This is supported by the following sub-themes, which emerged from this theme:

**Sub-theme 1.1: Knowledge of the importance of growth monitoring and promotion:** Knowledge of the importance of GMP is crucial and may help in motivating caregivers to adhere to sessions. Participants in this study reported knowledge of the importance of GMP, as supported by the following quotations:

‘GMP helps to identify growth faltering and developmental milestones in my child, so that immediate interventions can be brought.’ (Participant 14, 27 years, tertiary education, employed)

‘I know the importance of bringing the child to the clinic, the importance includes prevention against diarrhoea, infections, chicken pox and micronutrient deficiencies.’ *(Participant 19, 31 years, tertiary education, employed)*

On the contrary, other participants pointed out that they do not know the importance of GMP. The following quotations support the participants’ view:

‘I do not know the benefits of GMP and how it helps our children.’ (Participant 07, 44 years, secondary education, unemployed)

‘Honestly, I don’t know why scheduled clinic visits help our children; I hear others say it helps with growth of children, but I did not bring my first time to scheduled clinic and grew well.’ (Participant 11, 25 years, tertiary education, employed)

**Sub-theme 1.2: Lack of knowledge on the importance of adherence to growth monitoring and promotion sessions:** Adherence to the GMP sessions is beneficial to the health and growth of the child. It is important for caregivers to know importance of adherence to GMP sessions for them to consistently bring their children to all appointments or sessions. Participants in this study reported a lack of knowledge of the importance of adherence to GMP sessions, as evident from the following quotes:

‘I do not know the importance of adherence to GMP sessions; we sometimes come to clinic and not be assisted, that shows that it is not important to adhere to all GMP sessions.’ (Participant 04, 55 years, primary education, unemployed)

‘I do not know the benefits of GMP and the reasons for regular attendance, I just attend because they tell me so.’ (Participant 07, 44 years, secondary education, unemployed)

‘I don’t know the importance of adherence to GMP sessions, because I have not been bringing my first born consistently to clinic and he grew up well.’ (Participant 11, 25 years, tertiary education, employed)

**Sub-theme 1.3: Lack of knowledge on the interpretation of growth charts:** Participants in this study reported a lack of knowledge of growth charts and a lack of knowledge of the interpretation of growth-monitoring charts. The following statements from participants support this sub-theme:

‘I don’t know how to interpret the growth monitoring charts by myself, however, when I bring the child to clinic, I’m being told if the child is growing well or not.’ (Participant 09, 46 years, primary education, unemployed)

However, some participants indicated that they know how to interpret the growth-monitoring charts as supported by the following statements:

‘I do not know the benefits of GMP and the reasons for regular attendance, I just attend because they tell me so.’ (Participant 07, 44 years, secondary education, unemployed)

‘I don’t know the importance of adherence to GMP sessions, because I have not been bringing my first born consistently to clinic and he grew up well.’ (Participant 21, 44 years, tertiary education, unemployed)

#### Theme 2: Health services

The healthcare facilities provide GMP services to the communities, in particular caregivers of children under 5 years. How the GMP services are provided, including the provider of services, is crucial and may influence caregivers away from adherence to sessions. Participants in this study alluded that their adherence to GMP sessions is influenced by services rendered at healthcare facilities as supported by the following sub-themes:

**Sub-theme 2.1: Poor services by healthcare providers:** The quality of services provided at the healthcare facilities boost the trust and confidence the communities have in the facility, whilst poor services lead the communities, particularly caregivers, to lose trust in the healthcare. Participants in this study reported that poor services at the facility led them not to adhere to GMP sessions, as supported by the following quotations:

‘The fact that the clinic always lacks appropriate vaccines makes me and other mothers to not see the importance of GMP, thus we become demotivated.’ (Participant 02, 35 years, tertiary education, employed)

‘Sometimes we are told that the weighing scale at the clinic is not working, or the clinic has ran out of child vaccines/supplementation, as a results we end up not honouring appointments.’ (Participant 11, 25 years, tertiary education, employed)

**Sub-theme 2.2: Attitudes of healthcare providers:** Attitudes of healthcare providers give extra confidence in the facility; therefore, health care providers need to adopt cheerful, caring and positive attitudes whilst at work. Positive attitudes help in strengthening relationships and influence adherence to appointments. Participants in this study indicated non-adherence to GMP sessions because of poor attitudes of healthcare providers, as supported by the following quotation:

‘The nurses sometimes speak to us with disrespect, as if we chose for our children not to grow well; the disrespect demotivates us from adhering to sessions.’ (Participant 10, 18 years, secondary education, unemployed)

#### Theme 3: Socio-economic factors

The socio-economic profile of patients may determine their adherence to GMP sessions. Participants in this study indicated non-adherence to sessions because of work-related factors and lack of funds to travel to the healthcare facilities, as supported by the following sub-themes:

**Sub-theme 3.1: Work-related factors:** Some of the participants reported that they do not bring their children to the clinic as scheduled because they must go to work. At the same time, there are no adults at home to help in bringing the children to GMP sessions on their behalf, as supported by the following quotation:

‘I come to the clinic when I am not going to work as on other days the scheduled visits clash with my working days.’ (Participant 05, 23 years, secondary education, employed)

‘I sometimes miss the scheduled clinic visits because I have to go to work and do not have anyone at home to bring the child to the clinic for me.’ (Participant 22, 35 years, secondary education, employed)

**Sub-theme 3.2: Lack of transportation and lunch money:** Although primary health care facilities are meant to be easily accessed by communities, some of the community members require transportation to travel to their nearest clinic facilities. Therefore, this led to non-adherence because of lack of funds, as supported by the quotation below:

‘I sometimes not adhere to scheduled clinic visits or appointment because of lack of funds to travel to the clinic, including buying foods whilst at the clinic because we spend many hours at the clinic.’ (Participant 06, 37 Years, secondary education, unemployed)

## Discussion

Non-attendance of the scheduled clinic visits for GMP services have serious health repercussions for the children. Therefore, this study aimed to explore factors affecting non-adherence to GMP sessions amongst caregivers of children under five years in Polokwane. Participants in this study reported knowledge of the importance of GMP sessions and a lack of knowledge of the importance of adherence to GMP sessions. Participants further indicated inconsistent availability of resources permitting the provision of GMP services at healthcare facilities and non-adherence to GMP services with their firstborn children who nonetheless grew well as key factors influencing their view of adherence to GMP sessions as unimportant. According to WHO, access to essential medicines is one of the six building blocks for health systems.^17^ In the context of child health, access to GMP services is critical for the child’s health and development, including protection against diseases.^18^ The GMP is a critical technique to identify children under the age of 5 years whose growth does not keep up with expected patterns and that growth faltering is usually detected through regular weight measurement.^19^ Therefore, the inconsistent availability of GMP services would lead to delay in the detection of growth faltering and risk development of signs and symptoms of development of malnutrition. Growth monitoring charts are important tools used in infant and young child health, because they help to easily and quickly gain insights into early disease and the nutritional status of a child.^20^

A quantitative cross-sectional study conducted in Ghana reported that the majority of caregivers (93.7%) indicated that it is important to attend a GMP programme.^7^ Moreover, an Ethiopian study showed that more than half of caregivers understood the importance of regular GMP.^21^ Another study reported that caregivers honour clinic visits when told and not because they know the importance of adhering to the scheduled clinic visits,^22^ which report about 35.5% of caregivers adhered to clinic visits for the purpose of following the health professionals orders. Similar findings were reported in this study because participants indicated that they adhere to GMP sessions to comply with instructions of nurses, even though they do not know the importance of adherence to GMP sessions. The caregivers further mentioned that they adhered as the health professionals were the ones who know better than them.^22^ A Kenyan study indicated that most caregivers regard the main benefit of GMP as being vaccinating the children.^23^ In addition, participants alluded that they do not know the interpretation of growth charts. Knowledge or lack thereof may influence the adoption of particular health behaviour;^24^ in this context knowledge can influence adherence to GMP sessions. It was found that caregivers missed their children’s scheduled clinic appointments because of a lack of knowledge of the importance of attending sessions.^25^ Therefore, it is important for primary health care facilities to conduct a baseline analysis of the knowledge of the importance and overall goal of GMP amongst caregivers or target communities. Knowledge audits will help in the design and development of a clear educational intervention that addresses knowledge deficits which may otherwise influence non-adherence.

Participants in this study reported poor services at the healthcare facilities, which include long-waiting hours and attitudes of healthcare workers as contributing to non-adherence to GMP sessions. Similar findings were reported in various studies which indicated that poor services and long-waiting hours at the healthcare facilities significantly contribute to non-adherence.^26,27^ In addition, a Ghanian study reported the inability of healthcare providers to provide satisfactory education regarding the importance of GMP, influences caregivers to develop negative attitudes towards attendance of subsequent appointments.^8^ Similar findings were reported by participants in this study, who indicated that they sometimes see no value in adhering to clinic visits for their children to be weighed and told to go back and come back for another session. A cross-sectional study indicated that caregivers pointed out that healthcare workers fail to engage them in growth-monitoring procedures.^8^ Another study also indicated that healthcare workers do not discuss with caregivers relating to the growth and development of their children after weighing sessions and that they were not asked to interpret the growth chart either.^3^ It is important for healthcare providers at the facilities, particularly at the management level, to perform GMP service delivery audits and conduct an internal survey for clients to provide feedback on the GMP services to help them improve their services.

### Recommendations

This study recommends that the Department of Health (DOH) in Limpopo must prioritise and channelise resources to ensure availability of GMP services at all times. Moreover, there is a need to conduct knowledge assessment surveys amongst the communities and GMP services delivery audits and surveys to identify factors leading to non-adherence.

### Limitations

The findings cannot be extended to many caregivers of children under the age of five years in Limpopo; however, they are transferable to a different setting.

## Conclusion

Caregivers of children under the age of five years know the importance of GMP; however, they lack knowledge of the importance of adherence to GMP sessions. This study recommends conducting a knowledge assessment survey amongst caregivers to determine knowledge deficits regarding the importance of GMP and growth monitoring for the development of a clear educational intervention. Factors such as lack of knowledge of the importance of adherence to GMP sessions and long waiting hours at the clinic were found to be contributing enormously to non-adherence to GMP sessions. In addition, inconsistent availability of resources permitting the provision of GMP services at healthcare facilities and non-adherence to GMP services with their firstborn children who nonetheless grew well are key factors influencing not seeing the importance of adherence to GMP sessions. Therefore, it is recommended that the DOH must ensure availability of GMP services in all facilities to demonstrate the importance of and adherence to sessions. Experiences of caregivers with children who didn’t attend all GMP sessions who nonetheless grew well and healthy influence non-adherence. In addition, caregivers indicated that their socio-economic conditions such as lack of transportation money to the clinics, including lunch money, which is necessitated by long waiting hours at the clinics, contribute to non-adherence to GMP sessions. Therefore, there is a need for healthcare facilities to reduce waiting hours to minimise need for lunch money. Moreover, the primary health care providers should conduct service delivery audits and internal surveys for clients to provide feedback to identify factors that contribute to non-adherence in order to introduce measures to address them.
